# Light Triggers the Antiproliferative Activity of Naphthalimide-Conjugated (η^6^-arene)ruthenium(II) Complexes

**DOI:** 10.3390/ijms23147624

**Published:** 2022-07-10

**Authors:** Franco Bisceglie, Giorgio Pelosi, Nicolò Orsoni, Marianna Pioli, Mauro Carcelli, Paolo Pelagatti, Silvana Pinelli, Peter J. Sadler

**Affiliations:** 1 Department of Chemistry, Life Sciences and Environmental Sustainability, University of Parma, 43124 Parma, Italy; giorgio.pelosi@unipr.it (G.P.); orsoninicolo@gmail.com (N.O.); pioli.marianna@gmail.com (M.P.); mauro.carcelli@unipr.it (M.C.); paolo.pelagatti@unipr.it (P.P.); 2 C.O.M.T. Centre for Molecular and Translational Oncology, University of Parma, 43124 Parma, Italy; 3 Department of Medicine and Surgery, University of Parma, 43126 Parma, Italy; silvana.pinelli@unipr.it; 4 Department of Chemistry, University of Warwick, Gibbet Hill Road, Coventry CV4 7AL, UK; p.j.sadler@warwick.ac.uk

**Keywords:** Ru(II) arene complexes, photoactivation, photoactivated chemotherapy, naphthalimide

## Abstract

We report the synthesis and characterization of three half-sandwich Ru(II) arene complexes [(η^6^-arene)Ru(N,N′)L][PF_6_]_2_ containing arene = p-cymene, N,N′ = bipyridine, and L = pyridine meta- with methylenenaphthalimide (C1), methylene(nitro)naphthalimide (C2), or methylene(piperidinyl)naphthalimide (C3). The naphthalimide acts as an antenna for photoactivation. After 3 h of irradiation with blue light, the monodentate pyridyl ligand had almost completely dissociated from complex C3, which contains an electron donor on the naphthalimide ring, whereas only 50% dissociation was observed for C1 and C2. This correlates with the lower wavelength and strong absorption of C3 in this region of the spectrum (λ_max_ = 418 nm) compared with C1 and C2 (λ_max_ = 324 and 323 nm, respectively). All the complexes were relatively non-toxic towards A549 human lung cancer cells in the dark, but only complex C3 exhibited good photocytoxicity towards these cancer cells upon irradiation with blue light (IC_50_ = 10.55 ± 0.30 μM). Complex C3 has the potential for use in photoactivated chemotherapy (PACT).

## 1. Introduction

Naphthalimides (NIs) are a class of organic molecules characterized by a planar structure and a high electronic delocalization over the naphthalene moiety and the imide group [1]. They have been extensively studied for applications in medicinal chemistry because their planarity makes them good DNA intercalators [2,3]. Among them, Amonafide and Mitonafide have obtained FDA approval for the treatment of Secondary Acute Myeloid Leukemia, but unfortunately both present toxicity problems and side effects [4]. A key characteristic that makes NIs very appealing is the simplicity of their functionalization, which endows them with a coordinative ability that enables metal ion binding.

In the literature [5], several ruthenium(II) arene complexes have been reported that contain a monodentate pyridine tethered to the imide nitrogen of the NIs, and in these cases the metal coordination geometry is completed by two chloride ligands. The well-known fluorescent properties of NIs, combined with the potency of arene-Ru(II) complexes as antitumor compounds [6,7,8], pave the way for the synthesis of Ru-based photoactivatable chemotherapeutic compounds.

Photoactivated chemotherapeutic (PACT) compounds change their structures upon exposure to light [9,10,11], and this structural change leads to an enhancement of biological activity. In principle, an ideal PACT compound should be completely inactive in cells until it is photoactivated. In this way, a PACT compound can be selectively activated in a specific region of space at a specific time [12].

An interesting class of compounds in the PACT field are half-sandwich arene-Ru(II) complexes. A new series of piano stool complexes, with the general formula [(η^6^-arene)Ru(N,N′)L][PF_6_]_2_, containing a chelating ligand (N,N′) and a pyridine-type monodentate ligand (L) has recently been developed [13]. The uniqueness of this class of complexes is their ability to release L when irradiated with UVA light. The release of the ligand L leads to the formation of the aqua complex [(η^6^-arene)Ru(N,N′)(OH_2_)]^2+^, a reactive species that is effectively active in cells [14,15].

**Figure 1:**
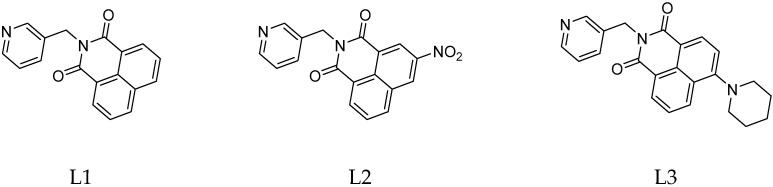
Structures of the ligands L1, L2, and L3.

In this paper, pursuing our ongoing interest to explore the photoactivation behavior and the biological activity of piano stool Ru(II) complexes [15], we have substituted the pyridine moiety with pyridine-functionalized NI ligands, obtaining three piano stool Ru(II) complexes with three different NIs as monodentate ligands (Figure 1); L1 is devoid of functionalization, L2 has a strong electron-withdrawing group in position 3, and L3 has a strong electron donating group in position 4. The choice of the functional groups is important, because the type of substituents and their position with respect to the amide are responsible for the different photophysical properties of the NIs [16].

The presence of a strong electron-withdrawing group, such as the nitro group (L2), leads to an increase in the energy of the excited states. On the other hand, the positioning of an electron-donor group in the *para* position with respect to the amide shifts the absorption maximum towards the blue region, with the formation of an intense band between 400 and 500 nm. This shift is due to a “push–pull” effect through an internal charge transfer.

**Figure 2:**
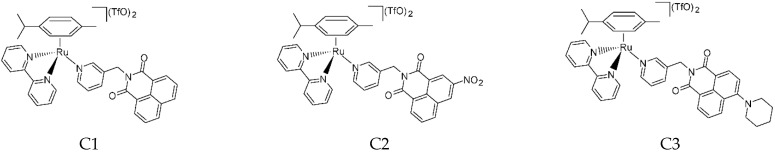
Structures of the complexes C1, C2, and C3.

Within this framework, we have prepared the new complexes C1–C3 (Figure 2).

## 2. Results

### 2.1. Synthetic Approach

**Scheme 1:**
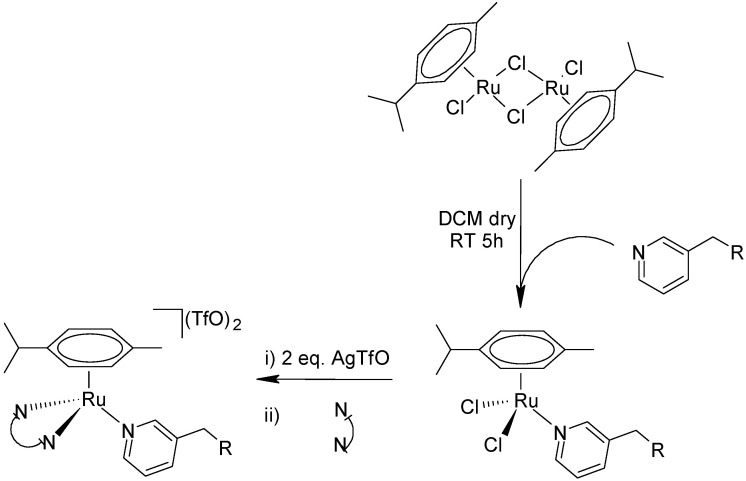
Synthetic route used for complexes C1–C3.

The complexes C1–C3 have been obtained through different synthetic pathways with respect to Sadler’s original strategy [13]. This synthetic route (Scheme 1) firstly involves the insertion of the monodentate ligand followed by the bidentate ligand. This inversion of order allows for the use of stoichiometric amounts of reagents, avoiding a large excess of the monodentate ligand (10–20 mol equivalent) that eventually leads to problems in the purification of the products as well as in the recovery of the unreacted ligand.

Two mol equivalents of the monodentate NI ligand were mixed with one mol equivalent of the ruthenium dimer [(η^6^-p-cymene)RuCl_2_]_2_ in dry dichloromethane under a nitrogen atmosphere. The intermediate neutral complex was subsequently mixed with two mol equivalents of silver triflate to remove the two chloride ligands. The so-formed highly unsaturated complex reacted quickly with the bidentate ligand (2,2′-bipyridine), leading to the final bi-cationic complex in which two triflate anions neutralized the positive charges of ruthenium without having interfered with the metal coordination. The three complexes C1, C2, and C3 were isolated in good yields and purity, as indicated by the elemental analyses, spectroscopic and spectrometric characterizations (Figures S1–S6).

### 2.2. Complex Stability

**Figure 3:**
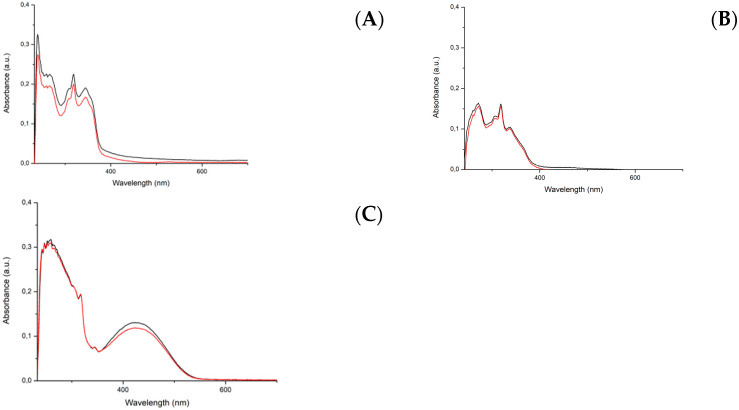
UV-vis studies of the stability of solutions of the complexes: (**A**) C1, (**B**) C2, and (**C**) C3, all 20 μM. Comparison between time-zero solution (black line) with the same solution after 24 h of incubation at 37.5 °C in the dark (red line).

The key feature that makes PACT compounds so interesting is their ability to release a ligand only when the complex is irradiated with the appropriate light wavelength. Stability in the dark, under physiological conditions, is a crucial prerequisite for the drug to be activated solely by light. The biological activity of C1, C2, and C3 was investigated by dissolving the target complex in DMSO and preparing a stock solution of a known concentration that was subsequently diluted in the cell culture medium. Hence, the stability was evaluated using a mixture of an aqueous phosphate buffered saline solution (PBS) with 5% of DMSO by comparing the UV spectrum of a complex solution, recorded immediately after its preparation, and that obtained after the storage of the same solution in the dark at 37.5 °C for 24 h; the two spectra were superimposable for all three complexes, thus confirming their stability in the DMSO/PBS mixture (Figure 3).

### 2.3. Absorption and Emission Spectra of the Ru(II) Complexes

**Figure 4:**
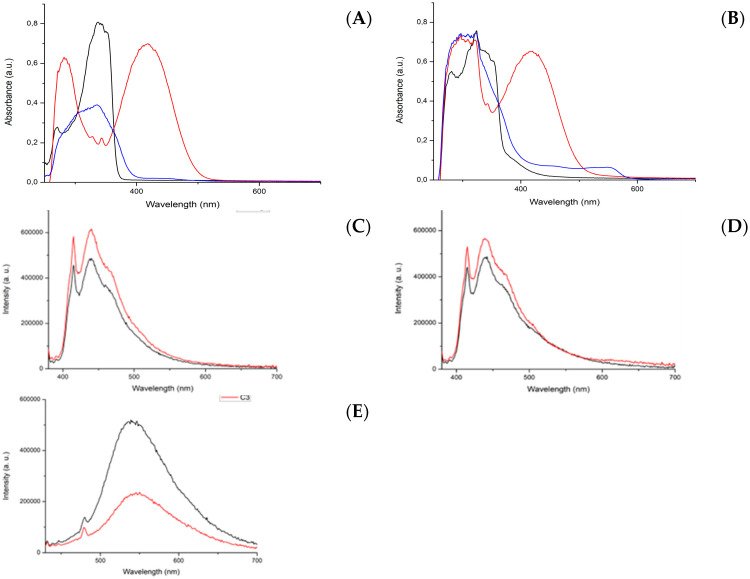
Absorption spectra recorded in DMSO at 100 μM of: (**A**) L1 (black line), L2 (blue line), and L3 (red line); (**B**) C1 (black line), C2 (blue line), and C3 (red line). Emission spectra in DMSO at 10 μM of: (**C**) L1 (black line) and C1 (red line), with λ_ex_ = 370 nm; (**D**) L2 (black line) and C2 (red line), with λ_ex_ = 370 nm; and (**E**) L3 (black line) and C3 (red line), with λ_ex_ = 418 nm.

The different functionalization of the naphthalene moiety (3-nitro-1,8-naphthalimide or 4-piperidinyl-1,8-naphthalimide) causes a shift of the absorption maxima in the electronic spectra of the free ligands. In Figure 4A, the absorption spectra of the three ligands are compared. L1 and L2 are characterized by a maximum absorption in the UV region, while L3, due to the “push–pull” effect, has a maximum in the blue region at 418 nm [17]. The absorption spectra of the three ruthenium complexes C1–C3 under the same conditions are compared in Figure 4B. Here, the maximum absorption corresponds with that of their related free NI ligands.

Next, we recorded the emission spectra of the complexes. Complexes C1 and C2, as well as their ligands L1 and L2, were excited with the same wavelength, 370 nm (UVA). For complex C3 and its ligand L3, which absorb in the visible region, we used an excitation wavelength of 418 nm (blue light).

In Figure 4C,D, the emission spectra of the two pairs L1/C1 and L2/C2 are compared under the same conditions. In both cases, the complexes have an emission band that is slightly more intense with respect to the ligand alone. For the pair L3/C3, the emission of the complex is significantly less intense than that of the ligand (Figure 4E).

This behavior can be ascribed to a radiationless mechanism of the deactivation of the excited state. The main spectroscopic properties of the ligands and complexes are listed in Table 1, where λ_em_ refers to an excitation wavelength of 370 nm, the molar extinction coefficient is based on the maximum absorption, and the quantum yield was obtained using quinine sulfate as a reference.

### 2.4. Photoactivation Experiments

Before studying the photoactivation of these Ru(II) complexes in cancer cells, ^1^H NMR was used to monitor time-dependent photodecomposition. The photoactivation of the complexes C1, C2, and C3 was investigated using a “photo-oven” built in the lab. The “photo-oven” consists of a plastic box with an internal light source, which can be regulated from the outside through a switch. A lid ensured that the box was completely dark when the internal light was switched off. The sample could be placed at a distance of 0 up to 25 cm from the light source and the temperature was monitored by a thermometer. No temperature modifications induced by the light source were registered during the experiments. The light source was a blue LED light centered at 420 nm, with a power of irradiance of 1.5 J/cm^2^ per hour. The same lamp was subsequently used for photoactivation of the cells.

**Scheme 2:**
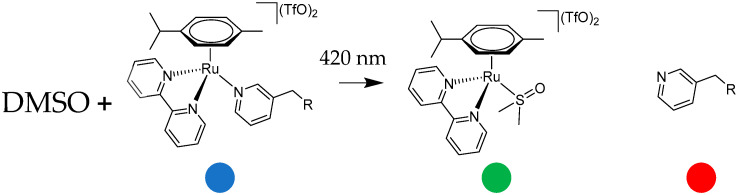
Photochemical decomposition of the complexes. The color code denotes assignment of peaks for the initial complex (blue) and the products (green and red) in the ^1^H NMR spectrum (Figure 5, Figure 6 and Figure 7).

**Figure 5:**
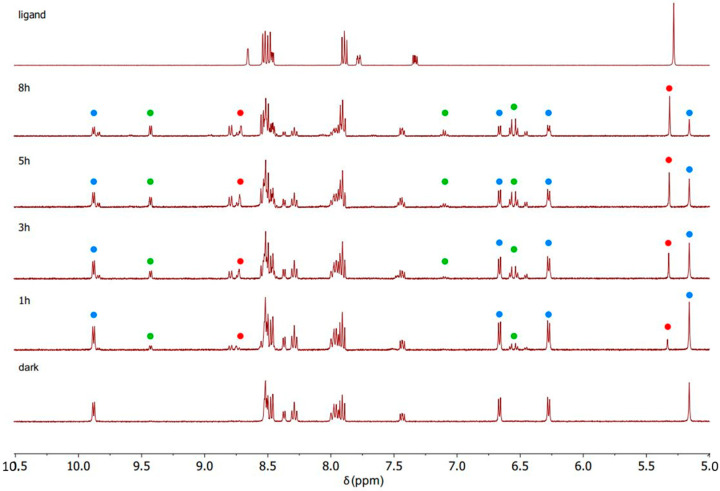
^1^H NMR spectra recorded during the photoactivation of complex C1 with blue light (420 nm) in DMSO-d_6_.

**Figure 6:**
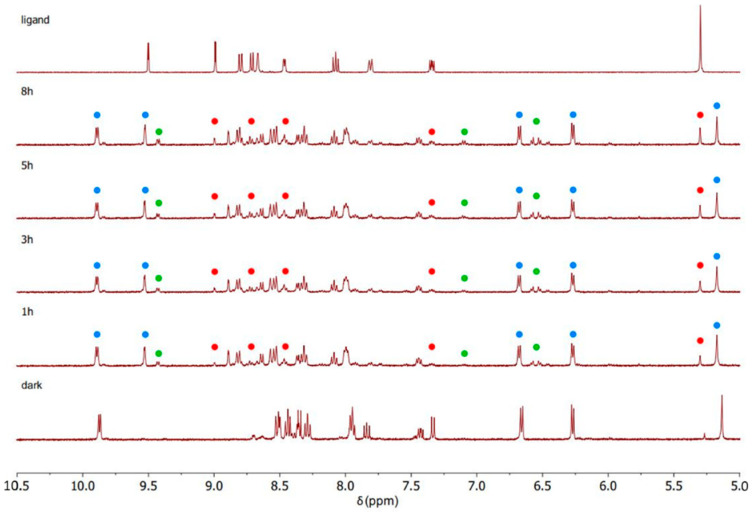
^1^H NMR spectra recorded during the photoactivation of complex C2 with blue light (420 nm) in DMSO-d_6_.

**Figure 7:**
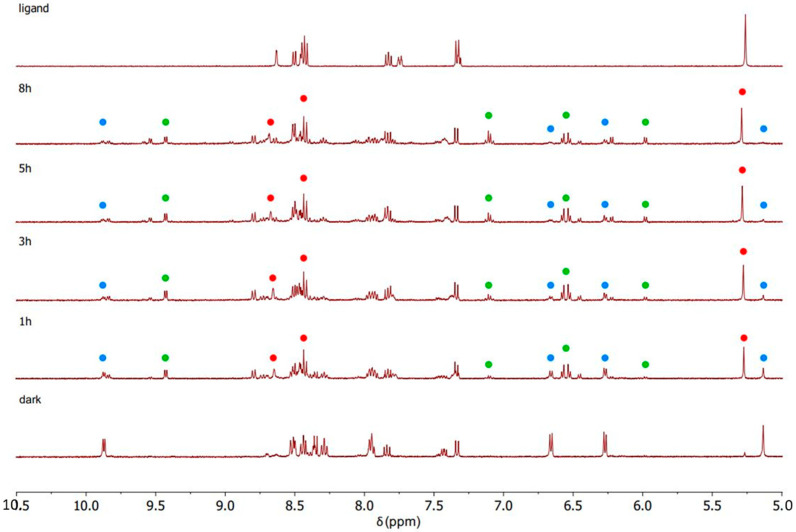
^1^H NMR spectra recorded during the photoactivation of complex C3 with blue light (420 nm) in DMSO-d_6_.

The complexes were dissolved in DMSO-d6 for photoactivation. This solvent was chosen to provide sufficient solubility within the concentration range required for the ^1^H NMR experiments. All the samples were prepared with the same concentration of 3 mM complex in 500 μL of DMSO-d_6_. The effects of the light irradiation were analyzed by collecting five spectra of the same sample: a “dark” spectrum, collected immediately after the sample preparation, and after 1, 3, 5, and 8 h of irradiation. Scheme 2 describes the photochemical decomposition pathway together with the color code used in Figure 5, Figure 6 and Figure 7 for the ^1^H NMR assignments.

C1–C3 were photoactivated by 420 nm blue LED light. During the irradiation, the intensity of the signals of the starting complex (the blue spots in Figure 5, Figure 6 and Figure 7) decreased and simultaneously new sets of signals appeared. These signals are associated with the two products of the photolysis: the free naphthalimide ligand (the red spots in Figure 5, Figure 6 and Figure 7) and [(η^6^-*p*-cym)Ru(N,N-bipy)(DMSO)]^2+^ (the green spots in Figure 5, Figure 6 and Figure 7). Details of the assignments can be found in the Materials and Methods section. The peaks for the released ligands match those of the free ligands, while the solvated ruthenium complex is detectable as a product by the doublet at 9.43 ppm, the signal of the bipyridine protons close to the nitrogen, and the quadruplet at 7.10 ppm, together with peaks close to 6.50 ppm from p-cymene. The ^1^H NMR spectra highlight the aromatic region since that is the more diagnostic part for observing the photoactivation.

A signal that is diagnostic for following the photolysis process is the methylene group singlet of the naphthalimide ligands (Figure S7), which shifts markedly when the naphthalimide is released via irradiation; therefore, this signal was used to study the kinetics of the photolysis (Table S1). All three complexes release the monodentate naphthalimide ligand when irradiated. The main distinction between the three complexes is the remarkable difference in the rates, as indicated by the time dependence of the singlet for the methylene group of the naphthalimides (Figure S7). After 3 h of irradiation, all of complex C3 had decomposed, but the corresponding amount for C1 and C2 was less than 50%.

### 2.5. Photocytotoxicity towards Cancer Cells

Next, we evaluated the cytotoxicity of the ruthenium complexes C1–C3 towards A549 human cancer cells with and without exposure to light. We chose cell line A549 as a target since this represents a solid lung tumor that currently has no effective cure, and that, at least in principle, is suitable for PACT treatments. The cytotoxicity was determined using the MTT assay, and then comparing cells treated in the dark with those receiving 3 h of light irradiation. The test consists of a 24 h treatment in which cells are exposed to the drug in the dark. Then the cells are exposed for 3 h to light irradiation and finally the cell viability is measured using the MTT assay.

We limited the exposure to 3 h because after longer exposures the cells began to suffer from the irradiation. In addition, to be sure to avoid the potentially harmful effects of light, as a control, we also irradiated cells not treated with the drug. The results are reported in Table 2.

The ligands L1, L2, and L3 alone were completely inactive, and this was probably due to their bulkiness with respect to the non-substituted naphthalimide. Planarity is important for strong intercalation, but all of these three ligands present potential steric hindrances, such as the presence of the pyridine linked to the imide moiety through the methylene fragment and the substituents on the aromatic rings [17]. Yet, all this notwithstanding, the naphthalimide ligands play a key role in the biological activity of the complexes, first because they act as antennae, and secondly because they may enhance the uptake of the Ru fragment into the cell. The previously synthesized Ru(II) complex [(η^6^-*p*-cym)Ru(N,N′-bpy)Cl](PF_6_) (C4) [15], for example, without naphthalimide ligands, is also completely inactive in cells after 3 h of irradiation (Table 2). C3 is the only complex that exhibits significant photocytotoxicity and the only compound able to absorb in the blue region. In addition, C3 is the only complex that, once irradiated, releases photoproducts with good kinetics (C3 is faster than C2, which is faster than C1). The two photoproducts are the monodentate NI ligand and the solvated ruthenium complex [(η^6^-*p*-cym)Ru(N,N′-bpy)(DMSO)]^2+^ (Scheme 2; green point). It is also interesting to compare the biological activity of C3 and C5, another Ru(II) complex recently studied in our lab (C5, [(η^6^-*p*-cym)Ru(N,N′-bpy)L](CF_3_SO_3_)_2_, L = *N*-4-formylpyridine-2-quinolinecarboxaldehydethiocarbohydrazone) [15]. The two IC_50_ values are identical after irradiation (10.55 ± 0.30 μM and 10.52 ± 1.95, respectively), and this is probably because the biological activity of the two Ru(II) complexes depends on the formation of the same photoproduct [(η^6^-*p*-cym)Ru(N,N′-bpy)(DMSO)]^2+^.

## 3. Conclusions

Three new piano stool Ru(II) arene complexes C1, C2, and C3 with different pyridyl-naphthalimides as monodentate ligands bearing electron-donating (piperidine) or electron-withdrawing (NO_2_) substituents on the naphthalenimido ring have been synthesized and characterized. These new Ru(II) complexes are stable in the dark in physiological conditions, but after irradiation with blue light they release a monodentate pyridyl ligand. The three Ru(II) complexes have different photodissociation rates related to their absorption maxima in the UV-vis spectra at 324, 323, and 418 nm for C1, C2, and C3, respectively. In the dark, the Ru(II) complexes are inactive towards A549 human lung cancer cells, while notably complex C3, bearing a 4-piperidinyl- substituent and exhibiting a strong absorption band at 418 nm, possesses a good photocytotoxicity against irradiation with blue light (IC_50_ = 10.55 ± 0.30 μM), demonstrating that the conjugated NIs play a key role as an antenna in the photoactivation of these complexes.

## 4. Materials and Methods

### 4.1. General Methods and Instruments

All reactants used were purchased from Sigma Aldrich (Milan, Italy). The 400 MHz ^1^H NMR and 100 MHz ^13^C NMR spectra were recorded on a Bruker Anova spectrometer (Billerica, MA, USA). ESI-MS analysis was performed using a Waters Acquity Ultraperformance ESI-MS spectrometer with Single Quadrupole Detector (Sesto San Giovanni, MI, Italy). Elemental analyses were obtained using a CHNS Thermo Fischer (Rodano, MI, Italy). UV–vis spectra were recorded on a Thermo Scientific Evolution 260 Bio instrument (Rodano, MI, Italy). The light intensity measurements at 420 nm were carried out with an Optical Power Meter (Newport 840-C, Monza, Italy) equipped with a Mod. 818-UV detector (active area 1 cm^2^).

**Figure 8:**
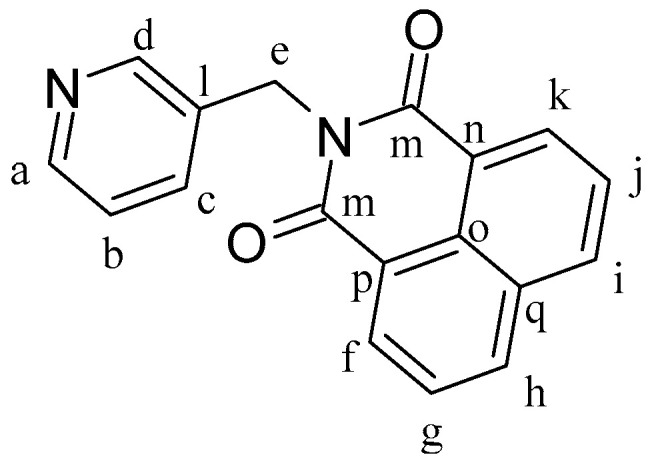
Structure of ligand ***N*-(3-methylenepyridyl)-1,8-naphthalimide (L1)**.

L1 (Figure 8) was synthesized according to a reported procedure [5]. 1,8-Naphthalenic anhydride (0.30 g, 1.51 mmol) and 3-picolylamine (0.196 g, 1.81 mmol) were dissolved in 10 mL of DMF, and the mixture was heated at 130 °C for 24 h. The mixture was cooled to room temperature, which yielded a pale yellow microcrystalline solid that was then separated by filtration and dried under vacuum. Yield: 87%. ^1^H-NMR (δ, ppm, 400 MHz, DMSO-d_6_): 8.66 (dd, J = 2.3 Hz, J′ = 0.9 Hz, 1H, H_d_), 8.51 (m, 4H, H_f_, H_h_, H_i_, H_k_), 8.46 (dd, J = 4.8 Hz, J′ = 1.7 Hz, 1H, H_a_), 7.89 (dd, J = 8.3 Hz, J′ = 7.3 Hz, 2H, H_b_, H_c_), 7.78 (ddd, J = 7.9 Hz, J′ = 2.4 Hz, J′′ = 1.7 Hz, 1H, H_g_), 7.33 (ddd, J = 7.9 Hz, J′ = 4.8 Hz, J′′ = 0.9 Hz, 1H, H_j_), 5.32 (s, 2H, H_e_). ^1^H-NMR (δ, ppm, 400 MHz, CDCl_3_): 8.89 (d, J = 2.1 Hz, 1H, H_d_), 8.62 (dd, J = 8.5 Hz, J = 1.0 Hz, 2H, H_f_, H_k_), 8.54 (dd, J = 5.2 Hz, J = 2.1 Hz, 1H, H_a_), 8.24 (dd, J = 8.5 Hz, J = 1.0 Hz, 2H, H_h_, H_i_), 8.02 (dt, J = 8.0 Hz, J′ = 2.1 Hz, 1H, H_b_), 7.76 (t, J = 8.5 Hz, 2H, H_g_, H_j_), 7.34 (ddd, J = 8.0 Hz, J′ = 5.2 Hz, J′′ = 2.1 Hz, 1H, H_c_), 5.43 (s, 2H, H_e_). ^13^C-NMR (δ, ppm, 100 MHz, DMSO-d_6_): 164.08 (C_m_), 149.75 and 148.83 (C_a,d_), 135.97 (C_f_), 135.12 (C_g_), 133.54 (C_b_), 131.84 (C_l_), 131.52 (C_h_), 128.01 (C_q_), 127.78 (C_n_), 124.05 (C_o_), 122.43 (C_c_), and 41.27 (C_e_). ESI-MS (+) m/z calc. 289.3, found 288.8. CHN analysis: C_18_H_12_N_2_O_2_: calculated: C: 74.99, H: 4.20, N: 9.72. Found: C: 74.93, H: 4.14, N: 9.80.

**Figure 9:**
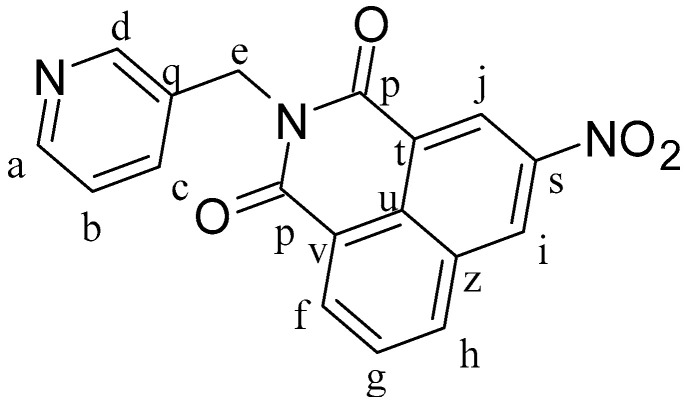
Structure of ligand **3-nitro-*N*-(3-methylenepyridyl)-1,8-naphthalimide (L2)**.

3-Nitro-1,8-naphthalenic anhydride (0.5 g, 2.06 mmol) and 3-picolylamine (0.267 g, 2.47 mmol) were dissolved in 25 mL of ethanol at 80 °C and heated for 24 h. After 1 h of reaction, there was a formation of a solid precipitate. The mixture was cooled to room temperature and a pale brown solid was separated by filtration and dried under vacuum, leading to the formation of L2 (Figure 9). Yield: 89%. ^1^H-NMR (δ, ppm, 400 MHz, DMSO-d_6_): 9.50 (d, J = 2.3 Hz, 1H, H_j_), 8.99 (d, J = 2.3 Hz, 1H, H_i_), 8.80 (dd, J = 8.4 Hz, J′ = 1.2 Hz, 1H, H_h_), 8.71 (dd, J = 7.3 Hz, J′ = 1.1 Hz, 1H, H_a_), 8.67 (d, J = 2.3 Hz, 1H, H_d_), 8.47 (dd, J = 4.9 Hz, J′ = 1.6 Hz, 1H, H_f_), 8.07 (dd, J = 8.3 Hz, J′ = 7.3 Hz, 1H, H_g_), 7.81 (dt, J = 7.9 Hz, J′ = 2.1 Hz, 1H, H_b_), 7.34 (m, 1H, H_c_), 5.30 (s, 2H, H_e_). ^1^H-NMR (δ, ppm, 400 MHz, CDCl_3_): 9.37 (d, J = 2.2 Hz, 1H, H_j_), 9.17 (d, J = 2.3 Hz, 1H, H_i_), 8.87 (d, J = 2.3 Hz, 1H, H_d_), 8.83 (dd, J = 7.3 Hz, J′ = 1.2 Hz, 1H, H_a_), 8.55 (dd, J = 4.8 Hz, J′ = 1.7 Hz, 1H, H_f_), 8.47 (ddd, J = 8.2 Hz, J′ = 1.2 Hz, J′′ = 0.5 Hz, 1H, H_h_), 7.98 (dd, J = 8.3 Hz, J′ = 7.3 Hz, 1H, H_g_), 7.93 (m, 1H, H_b_), 7.28 (m, 1H, H_c_), 5.43 (s, 2H, H_e_). ^13^C-NMR (δ, ppm, 100 MHz, CDCl_3_): 163.06 and 162.52 (C_p_), 149.49 (C_a_), 147.91 (C_s_), 146.36 (C_d_), 138.45 (C_j_), 136.02 (C_i_), 134.89 (C_h_), 133.11 (C_c_), 131.09 (C_q_), 130.12 (C_z_), 129.36 (C_g_), 129.23 (C_f_), 124.70 (C_z_), 124.43 (C_v_), 123.94 (C_t_), 122.92 (C_b_), and 41.41 (C_e_). ESI-MS (+) m/z calc. 334.30, found 334.15. CHN analysis: C_18_H_11_N_3_O_4_: calc.: C: 64.87, H: 3.33, N: 12.61. Found: C: 65.08, H: 3.29, N: 12.46.

**Figure 10:**
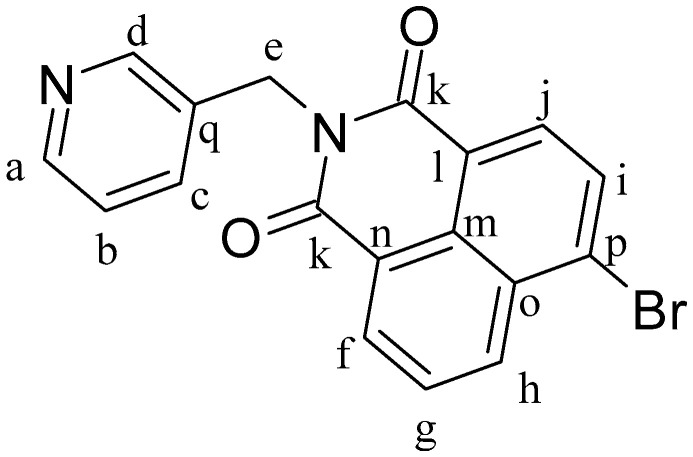
Structure of ligand **4-Bromo-*N*-(3-methylenepyridyl)-1,8-naphthalimide (L1Br)**.

4-Bromo-1,8-naphthalenic anhydride (0.2 g, 0.73 mmol) and 3-picolylamine (0.093 g, 0.86 mmol) were dissolved in 15 mL of ethanol at 80 °C and heated for 24 h. After 1 h of reaction, a precipitate formed. The mixture was cooled to room temperature and the white solid was separated by filtration and dried under vacuum, obtaining L1Br (Figure 10). Yield: 91%. ^1^H-NMR (δ, ppm, 400 MHz, CDCl_3_): 8.87 (d, J = 2.4 Hz, 1H, H_d_), 8.70 (dd, J = 7.3 Hz, J′ = 1.2 Hz, 1H, H_i_), 8.62 (dd, J = 8.5 Hz, J′ = 1.2 Hz, 1H, H_j_), 8.55 (dd, J = 5.0 Hz, J′ = 1.6 Hz, 1H, H_a_), 8.46 (d, J = 7.9 Hz, 1H, H_h_), 8.09 (d, J = 7.8 Hz, 1H, H_f_), 7.99 (dt, J = 8.0 Hz, J′ = 2.0 Hz, 1H, H_g_), 7.89 (dd, J = 8.5 Hz, J′ = 7.3 Hz, 1H, H_b_), 7.33 (dd, J = 8.0 Hz, J′ = 4.9 Hz, 1H, H_c_), 5.41 Hz (s, 2H, H_e_). ^13^C-NMR (δ, ppm, 100 MHz, CDCl_3_): 163.56 (C_k_), 150.25 and 148.56 (C_a_ and C_d_), 137.39 (C_f_), 133.70 (C_g_), 132.94 (C_p_), 132.44 (C_b_), 131.59 (C_h_), 131.22 (C_i_), 130.78 (C_q_), 130.71 (C_n_), 129.02 (C_o_), 128.18 (C_c_), 123.57 (C_j_), 122.79 (C_l_), 121.91 (C_m_), and 41.24 (C_e_). ESI-MS (+) m/z calc. 368.20, found 368.97. CHN analysis: C_18_H_11_BrN_2_O_2_: calculated: C: 58.88, H: 3.03, N: 7.63. Found: C: 58.84, H: 3.00, N: 7.88.

**Figure 11:**
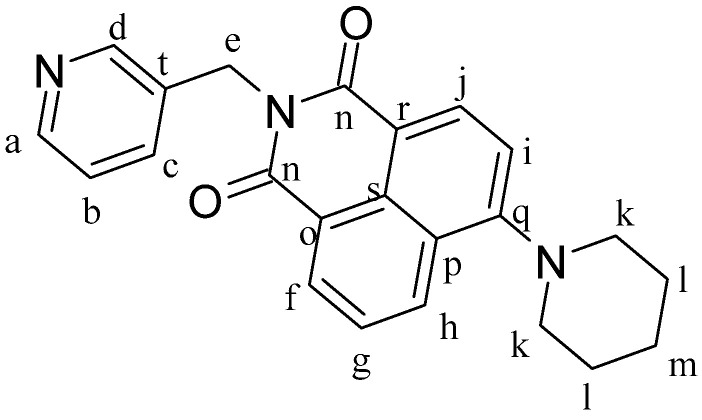
Structure of ligand **4-Piperidinyl-*N*-(3-methylenepyridyl)-1,8-naphthalimide (L3)**.

Ligand L3 (Figure 11) was synthesized following a procedure reported in the literature [18,19]. L1Br (0.459 g, 1.25 mmol) was dissolved in 3 mL of piperidine, and the mixture was heated to 110 °C for 2 h. After cooling to room temperature, the excess of piperidine was removed under vacuum. The solid was dissolved in hot ethanol and recrystallized at −4 °C. The product was then collected by filtration, washed with ethanol, and finally dried under vacuum. The process yielded a bright orange solid. Yield: 89%. ^1^H-NMR (δ, ppm, 400 MHz, DMSO-D_6_): 8.63 (d, J = 2.3 Hz, 1H, H_d_), 8.50 (dd, J = 7.3 Hz, J′ = 1.1 Hz, 1H, H_f_), 8.44 (m, 3H, H_a_, H_i_, H_j_), 7.83 (dd, J = 8.5 Hz, J′ = 7.3 Hz, 1H, H_b_), 7.74 (dt, J = 8.0 Hz, J′ = 2.0 Hz, 1H, H_g_), 7.33 (dd, J = 8.2 Hz, J′ = 5.4 Hz, 2H, H_h_, H_c_), 5.27 (s,2H, H_e_), 3.23 (d, J = 10.6 Hz, 4H, H_k_), 1.83 (bs, 4H, H_l_), 1.68 (bs, 2H, H_m_). ^1^H-NMR (δ, ppm, 400 MHz, CDCl_3_): 8.86 (d, J = 2 Hz, 1H, H_d_), 8.60 (dd, J = 8.4 Hz, J′ = 1.2 Hz, 1H, H_f_), 8.53 (dd, J = 8.4 Hz, J′ = 1.6 Hz, 2H, superimposed signals H_a_ e H_j_), 8.42 (d, J = 9.6 Hz, J′ = 1.2 Hz, 1H, H_h_), 7.99 (dt, J = 8 Hz, 1H, H_b_), 7.70 (dd, J = 16 Hz, J′ = 7.6 Hz, 1H, H_g_), 7.32 (dd, J = 12.8 Hz, J′ = 3,2 Hz, 1H, H_c_), 7.20 (d, J = 8.2 Hz, 1H, H_i_), 5.41 (s, 2H, H_e_), 3.26 (bs, 4H, H_k_),1.91 (bs, 4H, H_l_), and 1.75 (bs, 2H, H_m_). ESI-MS (+) m/z calc. 372.44, found 372.78. CHN analysis: C_23_H_21_N_3_O_2_: calc.: C: 74.37, H: 5.70, N: 11.31. Found: C: 74.15, H: 5.82, N: 11.24.

### 4.2. General Procedure for the Intermediate Complexes IC1, IC2, and IC3

**Figure 12:**
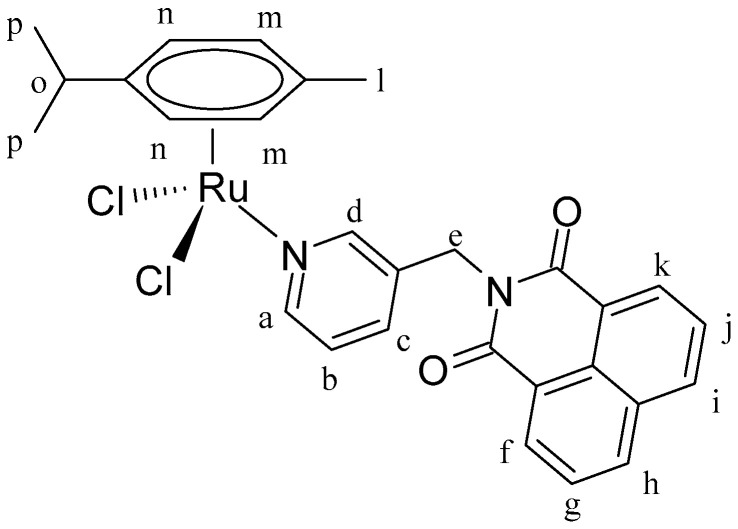
Structure of complex **[(η6-p-cym)Ru(L1)Cl2] (IC1)**.

**Figure 13:**
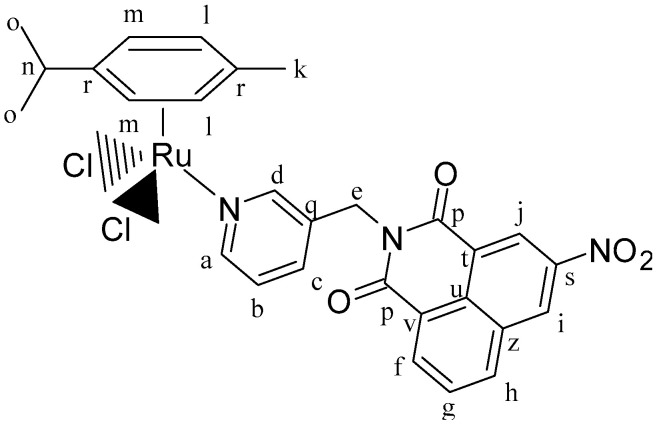
Structure of complex **[(η6-p-cym)Ru(L2)Cl2] (IC2)**.

**Figure 14:**
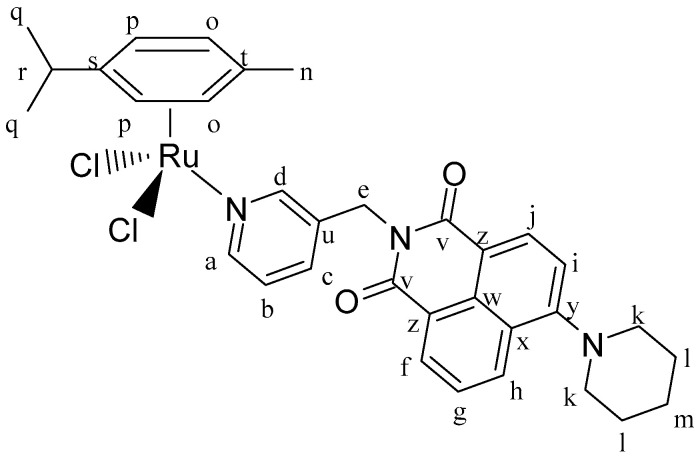
Structure of complex **[(η6-p-cym)Ru(L4)Cl2] (IC3)**.

[(η^6^-p-cym)RuCl_2_]_2_ (1 mol eq.) and the ligand (L1, L2, or L3, 2 mol eq.) were dissolved in CH_2_Cl_2_ dry in a Schlenk flask with nitrogen atmosphere. The mixture was stirred for 5 h, then diethyl ether was added, and the solution was left at −4 °C overnight. The precipitate was collected by filtration, washed with cold diethyl ether, and dried under vacuum, allowing to isolate complexes IC1 (Figure 12), IC2 (Figure 13) and IC3 (Figure 14).

[(η^6^-p-cym)RuCl_2_]_2_ (0.36 g, 1.26 mmol), L1 (0.38 g, 0.63 mmol). Yield: 83%. ^1^H-NMR (δ, ppm, 400 MHz, CDCl_3_): 9.36 (d, J = 2.0 Hz, 1H, H_d_), 8.95 (dd, J = 5.8, J′ = 1.4 Hz, 1H, H_a_), 8.66 (dd, J = 7.3, J′ = 1.1 Hz, 2H, H_f_, H_k_), 8.28 (dd, J = 7.3, J′ = 1.2 Hz, 2H, H_h_, H_i_), 7.95 (m, 1H, H_b_), 7.81 (dd, J = 8.2, J′ = 7.3 Hz, 2H, H_g_, H_j_), 7.25 (dd, J = 7.9, J′ = 5.7 Hz, 1H, H_c_), 5.46 (d, J = 5.9 Hz, 2H, H_n_), 5.39 (s, 2H, H_e_), 5.28 (d, J = 5.9 Hz, 2H, H_m_), 3.03 (m, 1H, H_o_), 2.15 (s, 3H, H_l_), 1.31 (d, J = 7.0 Hz, 6H, H_p_). ^13^C-NMR (δ, ppm, 100 MHz, CDCl_3_): 164.10, 156.83, 153.45, 138.43, 134.56, 134.21, 131.78, 131.72, 128.31, 127.16, 124.19, 122.39,103.77, 97.07, 82.74, 82.67, 53.49, 40.87, 30.68, 22.32, and 18.13. ESI-MS (+) m/z calc. 559.04, found 559.16. CHN analysis: C_28_H_26_Cl_2_N_2_O_2_Ru: calc.: C: 56.57, H: 4.41, N: 4.71. Found: C: 56.74, H: 4.30, N: 5.03.

[(η^6^-p-cym)RuCl_2_]_2_ (0.15 g, 0.24 mmol), L2 (0.16 g, 0.48 mmol). Orange solid. Yield: 85%. ^1^H-NMR (δ, ppm, 400 MHz, CDCl_3_): 9.35 (d, J = 2.1 Hz, 2H, H_d_, H_j_), 9.18 (d, J = 2.2 Hz, 1H, H_i_), 8.97 (d, J = 5.7 Hz, 1H, H_a_), 8.83 (d, J = 5.7 Hz, 1H, H_f_), 8.47 (d, J = 8.3 Hz, 1H, H_h_), 7.98 (m, 2H, H_b_, H_g_), 7.26 (s, 1H, H_c_), 5.46 (d, J = 5.8 Hz, 2H, H_m_), 5.40 (s, 2H, H_e_), 5.28 (d, J = 5.8 Hz, 2H, H_l_), 3.03 (m, 1H, H_n_), 2.16 (s, 3H, H_k_), 1.33 (d, J = 6.9 Hz, 6H, H_o_). ^13^C-NMR (δ, ppm, 100 MHz, CDCl_3_): 163.25 (C_p_), 162.34 (C_p_), 156.94 (C_a_), 153.43 (C_d_), 148.13 (C_s_), 138.94 (C_f_), 138.35 (C_b_), 136.98 (C_g_), 134.73 (C_h_), 134.52 (C_c_), 130.57 (C_t_), 129.86 (C_q_), 129.12 (C_i_), 129.04 (C_j_), 124.83 (C_u_), 124.21 (C_v_), 124.01 (C_z_), 103.69 (C_r_), 96.99 (C_r_), 82.63 (C_m,l_), 40.74 (C_e_), 30.58 (C_k_), 22.26 (C_o_), and 18.07 (C_n_). ESI-MS (+) m/z [M-Cl]^+^ calc. 604.05, found 604.22. CHN analysis: C_28_H_25_Cl_2_N_3_O_4_Ru: calc.: C: 52.59, H: 3.94, N: 6.57. Found: C: 52.26, H: 3.93, N: 6.26.

[(η^6^-p-cym)RuCl_2_]_2_ (0.10 g, 0.16 mmol), L3 (0.12 g, 0.32 mmol). Orange solid. Yield: 89%. ^1^H-NMR (δ, ppm, 400 MHz, CDCl_3_): 9.34 (d, J = 1.9 Hz, 1H, H_d_), 8.93 (dd, J = 5.6, J′ = 1.4 Hz, 1H, H_a_), 8.61 (dd, J = 7.3, J′ = 1.2 Hz, 1H, H_f_), 8.53 (d, J = 8.1 Hz, 1H, H_j_), 8.43 (dd, J = 8.5, J′ = 1.2 Hz, 1H, H_h_), 7.94 (dt, J = 7.9, J′ = 1.7 Hz, 1H, H_b_), 7.71 (dd, J = 8.5, J′ = 7.3 Hz, 1H, H_g_), 7.22 (m, 2H, H_c_, H_i_), 5.46 (d, J = 5.9 Hz, 2H, H_p_), 5.37 (s, 2H, H_e_), 5.28 (d, J = 5.9 Hz, 2H, H_o_), 3.27 (m, 4H, H_k_), 3.00 (m, 1H, H_r_), 2.13 (s, 3H, H_n_), 1.90 (m, 4H, H_l_), 1.76 (d, J = 5.8 Hz, 2H, H_m_), 1.30 (d, J = 6.9 Hz, 6H, H_q_). ^13^C-NMR (δ, ppm, 100 MHz, CDCl_3_): 164.48 (C_v_), 163.92 (C_v_), 157.82 (C_y_), 156.79 (C_a_), 153.13 (C_d_), 138.31 (C_b_), 134.47 (C_c_), 133.15 (C_w_), 131.40 (C_f_), 131.23 (C_g_), 130.07 (C_u_), 126.26 (C_x_), 125.44 (C_h_), 124.11 (C_i_), 122.71 (C_z_), 115.23 (C_z_), 114.79 (C_j_), 103.62 (C_s_), 97.03 (C_t_), 82.63 (C_o,p_), 54.56 (C_k_), 40.60 (C_e_), 30.61 (C_n_), 26.21 (C_k,l_), 24.32 (C_m_), 22.27 (C_q_), and 18.07 (C_r_). ESI-MS (+) m/z [M-Cl]^+^ calc. 642.18, found 642.13. CHN analysis: C_33_H_35_Cl_2_N_3_O_2_Ru calc.: C: 58.49, H: 5.21, N: 10.46. Found: C: 58.62, H: 5.04, N: 10.26.

### 4.3. General Procedure for Synthesis of Complexes C1, C2 and C3

**Figure 15:**
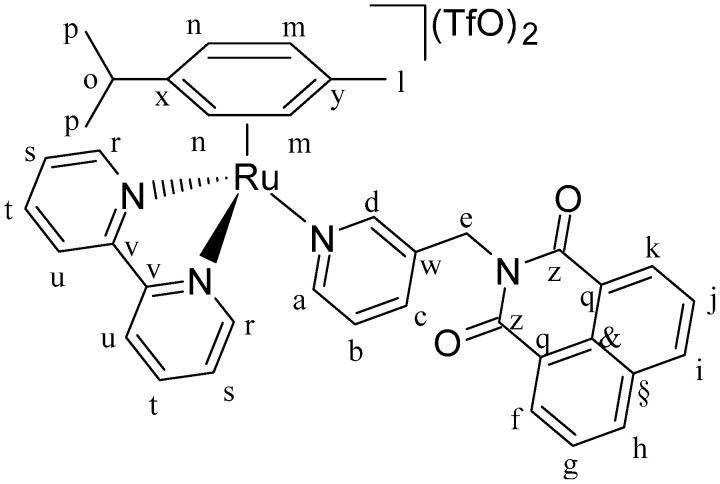
Structure of complex **[(η6-p-cym)Ru(N,N’-bpy)(L1)](SO3CF3)2 (C1)**.

**Figure 16:**
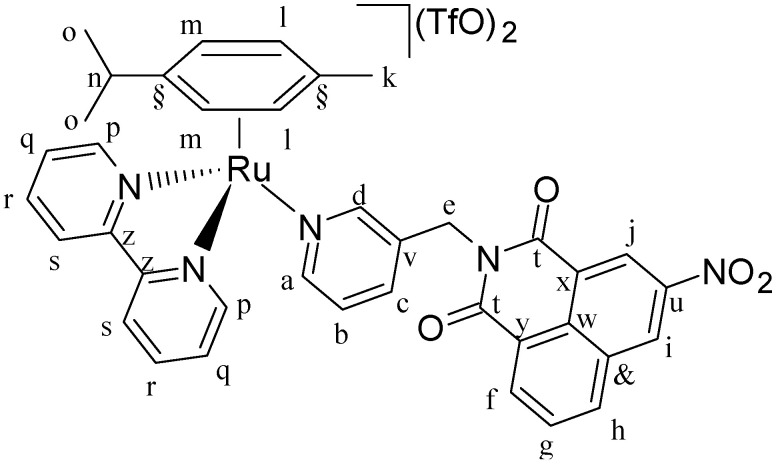
Structure of complex **[(η6-p-cym)Ru(N,N′-bpy)(L2)](SO3CF3)2 (C2)**.

**Figure 17:**
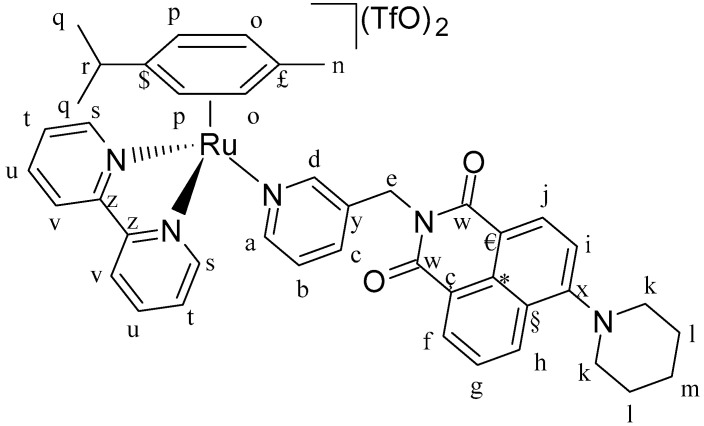
Structure of complex **[(η6-p-cym)Ru(N,N′-bpy)(L4)](SO3CF3)2 (C3)**.

The intermediate complex (IC1, IC2, or IC3, 1 eq.) was dissolved in dry CH_2_Cl_2_ in a Schlenk flask with nitrogen atmosphere. AgTfO (TfO = trifluoromethanesulfonate; 2 mol eq.) was dissolved in dry MeOH in nitrogen atmosphere and dropped slowly into the ruthenium complex solution. The solution became turbid immediately. It was stirred for 2 h at room temperature, and the flask was covered with aluminium foil to keep the solution in dark conditions. Then, the AgCl formed was removed by filtering with a Schlenk filter and the clear solution was dropped into a Schlenk flask containing bipyridine (1 mol eq.). The mixture was stirred overnight, always under a nitrogen atmosphere and in a dark environment. Next, diethyl ether was added, and the solution was left at −4 °C overnight. The precipitate was collected by filtration, washed with cold diethyl ether, and dried under vacuum. In this way complexes C1 (Figure 15), C2 (Figure 16) and C3 (Figure 17) were isolated.

IC1 (0.10 g, 0.17 mmol, 1 eq.), AgTfO (0.09 g, 0.34 mmol, 2 eq.) and 2,2′-bipyridine (0.03 g, 0.17 mmol, 1 eq.). Orange solid. Yield: 77%. ^1^H-NMR (δ, ppm, 400 MHz, DMSO-d_6_): 9.88 (dd, J = 5.7, J′ = 1.4 Hz, 2H, H_r_), 8.51 (ddd, J = 8.3, J′ = 3.7, J′′ = 1.3 Hz, 5H, H_a_, H_u_, H_f_, H_k_), 8.46 (d, J = 1.1 Hz, 1H, H_d_), 8.37 (d, J = 5.5 Hz, 1H, H_c_), 8.29 (td, J = 7.9, J = 1.4 Hz, 2H, H_s_), 7.96 (m, 4H, H_j_, H_i_, H_h_, H_g_), 7.91 (dd, J = 8.2, J′ = 7.3 Hz, 2H, H_t_), 7.43 (dd, J = 7.9, J′ = 5.7 Hz, 1H, H_b_), 6.66 (d, J = 6.3 Hz, 2H, H_m_), 6.27 (d, J = 6.3 Hz, 2H, H_n_), 5.16 (s, 2H, H_e_), 2.41 (m, 1H, H_n_), 1.78 (s, 3H, H_l_), 0.82 (d, J = 6.9 Hz, 6H, H_p_). ^1^H-NMR (δ, ppm, 400 MHz, CDCl_3_): 10.32 (d, J = 5.5 Hz, 2H, H_r_), 8.76 (d, J = 5.7 Hz, 1H, H_a_), 8.63 (s, 1H, H_d_), 8.50 (d, J = 7.2 Hz, 2H, H_u_), 8.25 (d, J = 8.4 Hz, 2H, H_k_, H_f_), 8.21 (t, J = 7.8 Hz, 2H, H_s_), 8.11 (m, 4H, H_t_, H_h_, H_i_), 7.93 (d, J = 9.0 Hz, 1H, H_c_), 7.79 (t, J = 8 Hz, H_g_, H_j_), 7.42 (t, J = 7.4 Hz, 1H, H_b_), 6.54 (d, J = 6.3 Hz, 2H, H_m_), 6.45 (d, J = 6.3 Hz, 2H, H_n_), 5.26 (s, 2H, H_e_), 2.42 (m, 1H, H_o_), 1.87 (s, 3H, H_l_), 0.93 (d, J = 6.8 Hz, 6H, H_p_). ^13^C-NMR (δ, ppm, 100 MHz, CDCl_3_): 163.71 (C_z_), 158.05 (C_r_), 156.38 (C_r_), 153.18 (C_a,d_), 140.63 (C_v_), 138.42 (C_b_), 134.81 (C_f,k_), 133.70 (C_q_), 131.86 (C_g,j_), 130.62 (C_w_), 127.09 (C_h,i_), 123.44 (C_c_), 121.87 (C_§,&_), 102.70 (C_x_), 92.36 (C_y_), 85.16 (C_m,n_), 40.30 (C_e_), 31.05 (C_l_), 22.27 (C_p_), and 18.03 (C_o_). ESI-MS(+) m/z [M-TfO^-^]^+^ m/z calc. 829.84 found 829.14. CHNS analysis: C_40_H_34_F_6_N_4_O_8_RuS_2_: calc.: C: 49.13, H: 3.50, N: 5.73, S: 6.56. Found: C: 48.81, H: 3.87, N: 5.75, S: 6.43.

IC2 (0.050 g, 0.078 mmol, 1 eq.), AgTfO (0.040 g, 0.156 mmol, 2 eq.) and 2,2′-bipyridine (0.012 g, 0.078 mmol, 1 eq.). Red solid. Yield: 71%. ^1^H-NMR (δ, ppm, 400 MHz, DMSO-d_6_): 9.89 (d, J = 6.2 Hz, 1H, H_p_), 9.53 (s, 1H, H_j_), 8.89 (s, 1H, H_i_), 8.82 (d, J = 8.1 Hz, 1H, H_a_), 8.64 (d, J = 7.4 Hz, 1H, H_f_), 8.57 (s, 1H, H_d_), 8.54 (d, J = 9.0 Hz, 2H, H_s_), 8.37 (d, J = 5.5 Hz, 1H, H_c_), 8.32 (t, J = 6.2 Hz, 2H, H_q_), 8.09 (t, J = 8.1 Hz, 1H, H_g_), 8.00 (s, 3H, H_r_, H_h_), 7.44 (t, J = 7.4 Hz, 1H, H_b_), 6.68 (d, J = 6.3 Hz, 2H, H_l_), 6.27 (d, J = 6.3 Hz, 2H, H_m_), 5.17 (s, 2H, H_e_), 2.45 (m, 1H, H_n_), 1.77 (s, 3H, H_k_), 0.81 (d, J = 6.8 Hz, 6H, H_o_). ^1^H-NMR (δ, ppm, 400 MHz, CDCl_3_): 10.40 (d, J = 5.6 Hz, 2H, H_p_), 9.14 (s, 1H, H_j_), 9.06 (s, 1H, H_i_), 8.78 (s, 2H, H_s_), 8.72 (d, J = 8.1 Hz, 1H, H_a_), 8.45 (d, J = 7.4 Hz, 1H, H_f_), 8.27 (t, J = 6.2 Hz, 2H, H_q_), 8.18 (t, J = 6.2 Hz, 2H, H_r_), 8.12 (d, J = 5.5 Hz, 2H, H_d_, H_h_), 7.98 (t, J = 8.1 Hz, 1H, H_g_), 7.90 (t, J = 5.5 Hz, 1H, H_c_), 7.43 (t, J = 7.4 Hz, 1H, H_b_), 6.50 (m, 4H, H_l_, H_m_), 5.34 (s, 2H, H_e_), 2.43 (m, 1H, H_n_), 1.90 (s, 3H, H_k_), 0.94 (d, J = 6.8 Hz, 6H, H_o_). ^13^C-NMR (δ, ppm, 100 MHz, CDCl_3_): 162.45 (C_t_), 162.15 (C_t_), 158.21 (C_p_), 154.51 (C_a_), 154.01 (C_d_), 146.68 (C_u_), 140.69 (C_z_), 140.09 (C_u_), 136.51 (C_f_), 136.33 (C_g_), 134.87 (C_h_), 131.15 (C_b,c_), 130.77 (C_w_), 130.43 (C_q_), 129.48 (C_v_), 129.33 (C_i_), 127.47 (C_j_), 124.24 (C_x_), 123.99 (C_y_), 123.85 (C_&_), 123.09 (C_r,s_), 103.98 (C_§_), 92.35 (C_m_), 84.85 (C_l_), 40.63 (C_e_), 31.12 (C_k_), 22.36 (C_o_), 21.93 (C_o_), and 18.05 (C_n_). ESI-MS (+) m/z [M-TfO^-^]^+^ m/z calc. 873.85. found 874.23. CHNS analysis: C_40_H_33_F_6_N_5_O_10_RuS_2_: calc.: C: 46.97, H: 3.25, N: 6.85, S: 6.27. Found: C: 46.52, H: 3.41, N: 6.30, S: 6.26.

IC3 (0.12 g, 0.17 mmol, 1 eq.), AgTfO (0.09 g, 0.35 mmol, 2 eq.) and 2,2′-bipyridine (0.03 g, 0.17mmol, 1 eq.). Orange solid. Yield: 91%. ^1^H-NMR (δ, ppm, 400 MHz, DMSO-d_6_): 9.87 (d, J = 5.5 Hz, 2H, H_s_), 8.51 (m, 3H, H_a_, H_d_, H_f_), 8.44 (dd, J = 7.8, J = 6.4 Hz, 2H, H_t_), 8.35 (m, 2H, H_j_, H_h_), 8.29 (t, J = 7.9 Hz, 2H, H_u_), 7.95 (t, J = 7.0 Hz, 3H, H_v_, H_c_), 7.84 (m, 1H, H_g_), 7.43 (m, 1H, H_b_), 7.33 (d, J = 8.3 Hz, 1H, H_i_), 6.66 (d, J = 6.2 Hz, 2H, H_p_), 6.27 (d, J = 6.3 Hz, 2H, H_o_), 5.13 (s, 2H, H_e_), 3.24 (s, 4H, H_k_), 2.45 (m, 1H, H_r_), 1.84 (s, 4H, H_l_), 1.78 (s, 3H, H_n_), 1.69 (s, 2H, H_m_), 0.82 (d, J = 6.9 Hz, 6H, H_q_). ^1^H-NMR (δ, ppm, 400 MHz, CDCl_3_): 10.24 (d, J = 5.7 Hz, 2H, H_s_), 8.76 (d, J = 5.5 Hz, 1H, H_a_), 8.56 (s, 1H, H_d_), 8.41 (t, J = 6.2 Hz, 2H, H_t_), 8.35 (d, J = 8.1 Hz, 1H, H_f_), 8.20 (m, 4H, H_v_, H_h_, H_j_), 8.07 (t, J = 6.2 Hz, 2H, H_u_), 7.93 (d, J = 7.4 Hz, 1H, H_c_), 7.69 (d, J = 8.5, 1H, H_g_), 7.40 (t, J = 7.4 Hz, 1H, H_b_), 7.24 (d, J = 8.2 Hz, 1H, H_i_), 6.52 (d, J = 6.3 Hz, 2H, H_o_), 6.42 (d, J = 6.3 Hz, 2H, H_p_), 5.20 (s, 2H, H_e_), 3.29 (s, 4H, H_k_), 2.41 (m, 1H, H_r_), 1.92 (bs, 4H, H_l_), 1.86 (s, 3H, H_n_), 1.77 (s, 2H, H_m_), 0.92 (d, J = 6.8 Hz, 6H, H_q_). ^13^C-NMR (δ, ppm, 100 MHz, CDCl_3_): 163.91 (C_w_), 163.05 (C_w_), 157.35 (C_x_), 154.78 (C_s_), 153.04 (C_a_), 140.68 (C_z_), 140.39 (C_d_), 136.81 (C_b_), 133.04 (C_c_), 131.55 (C_f_), 131.07 (C_g_), 130.42 (C_€_), 130.35 (C_t_), 129.47 (C_y_), 127.35 (C_u_), 125.73 (C_ç_), 125.31 (C_h_), 123.67 (C_v_), 122.36 (C_i_), 122.11 (C_*_), 119.26 (C_§_), 116.00 (C_j_), 109.26 (C_£_), 103.45 (C_$_), 92.42 (C_o_), 85.00 (C_p_), 55.21 (C_k_), 40.23 (C_e_), 31.24 (C_n_), 25.75 (C_l_), 23.66 (C_m_), 22.19 (C_q_), and 17.94 (C_q_). ESI-MS (+) m/z [M-TfO^-^]^+^ m/z calc. 911.98. found 912.49. CHNS analysis: C_45_H_43_F_6_N_5_O_8_RuS_2_: calc.: C: 50.94, H: 4.09, N: 6.60, S: 6.04. Found: C: 50.86, H: 4.05, N: 6.34, S: 5.99.

### 4.4. Cell Biology

#### 4.4.1. Cell Lines

The A549 human lung cancer cells were purchased from the American Type Culture Collection (ATCC, Manassas, VA, USA). Cells were grown in RPMI 1640 medium containing 10% heat-inactivated fetal bovine serum, 100 U mL^−^^1^ penicillin, and streptomycin at 37 °C with 5% CO_2_. Experiments were conducted with cells in the log phase and A549 adherent cells were harvested using trypsin.

#### 4.4.2. Cell Viability Assay

Drug effects on cell viability were analyzed using a 3-(4,5-dimethylthiazol-2-yl)-2,5-diphenyltetrazolium bromide (MTT) colorimetric assay, based on the ability of the metabolically active cells to reduce MTT to formazan by the action of mitochondrial dehydrogenases. A549 (70,000 mL^−1^) cells were seeded into 96-well plates overnight, and then exposed to compounds at indicated concentrations. At the end of the treatment, MTT was added (final concentration 0.5 mg mL^−1^) for 3 h at 37 °C and formazan crystals were dissolved in 100 mL for each well of acidic isopropanol (0.08 N HCl). After mixing, the absorbance was determined using a Multiskan Ascent microwell plate reader equipped with a 550 nm filter (Thermo Labsystems, Helsinki, Finland). At least three independent experiments were performed with eight replicate wells per sample. The half maximal inhibitory concentration (IC_50_) was determined as the concentration resulting in 50% cell growth reduction compared with untreated control cells.

## Figures and Tables

**Table 1:** Spectroscopic properties of ligands and complexes.

Compound	λ_abs_ (nm)	λ_em_ (nm)	ε(M^−1^ cm^−1^)	Φf
L1	337	440	7685	0.046
C1	324	440	6392	0.030
L2	336	440	3517	0.051
C2	323	440	7121	0.225
L3	418	550	7020	0.060
C3	418	550	6543	0.027

**Table 2:** Cytotoxicity of the ligands L1, L2, and L3, and the complexes C1, C2, C3, C4, and C5 (IC_50_ values) towards A549 human lung cancer cells after incubation for 24 h in the absence (dark) and presence of blue light irradiation for 3 h (λ = 420 nm, power 4.5 J/cm^2^). The IC_50_ of [(η^6^-*p*-cym)Ru(N,N′-bpy)Cl](PF_6_) (C4) and [(η^6^-*p*-cym)Ru(N,N′-bpy)L](SO_3_CF_3_)_2_ (L = *N*-4-formylpyridine-2-quinolinecarboxaldehydethiocarbohydrazone) (C5) are reported for comparison.

Compound	IC_50_ (μM) A549 (24 h)	
	Dark	3 h of Irradiation *
L1	>100	>100
C1	>100	>100
L2	>100	>100
C2	>100	>100
L3	98 ± 6	>100
C3	76.7 ± 1.3	10.5 ± 0.3
C4	>100	>100
C5	84.6 ± 3.9	10.5 ± 1.9

* The test consists of a 24 h treatment in which cells are exposed to the drug in the dark. Then the cells are exposed for 3 h to light irradiation and finally the cell viability using MTT was measured.

## Data Availability

Not applicable.

## Supplementary Materials

The following supporting information can be downloaded at: https://www.mdpi.com/article/10.3390/ijms23147624/s1.
