# Supplementary material for: Light Triggers the Antiproliferative Activity of Naphthalimide-Conjugated (η6-arene)ruthenium(II) Complexes
Source: Int J Mol Sci. 2022 Jul 10;23(14):7624. doi: 10.3390/ijms23147624 (PMC9322830; doi:10.3390/ijms23147624)
Supplement: Supplementary file 1 [file ijms-23-07624-s001.zip › ijms-1774491-supplementary.pdf]

Supporting Information

Light Triggers the Antiproliferative Activity of Naphthalimide-Conjugated (η^6-arene)ruthenium(II) Complexes

Franco Bisceglie [1,2,*], Giorgio Pelosi [1,2], Nicolò Orsoni [1], Marianna Pioli [1], Mauro Carcelli [1], Paolo Pelagatti [1], Silvana Pinelli [3] and Peter J. Sadler [4]

Table S1. Chemical shift of the methylene group in the complexes and their ligands.

	C	L
**C1/L1**	5.16 ppm	5.32 ppm
**C2/L2**	5.17 ppm	5.30 ppm
**C3/L4**	5.13 ppm	5.27 ppm

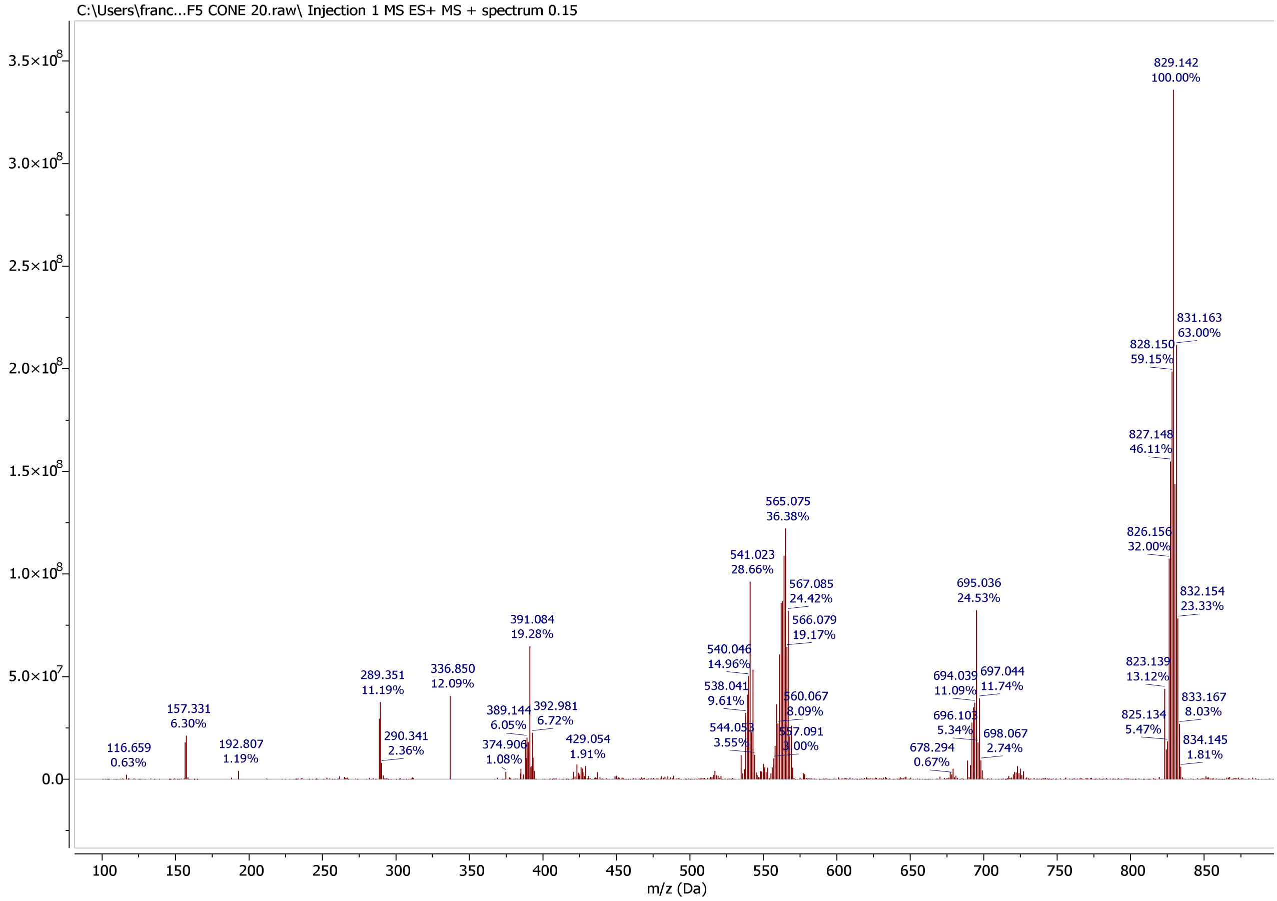

Figure S1. Full ESI mass spectrum of compound C1.

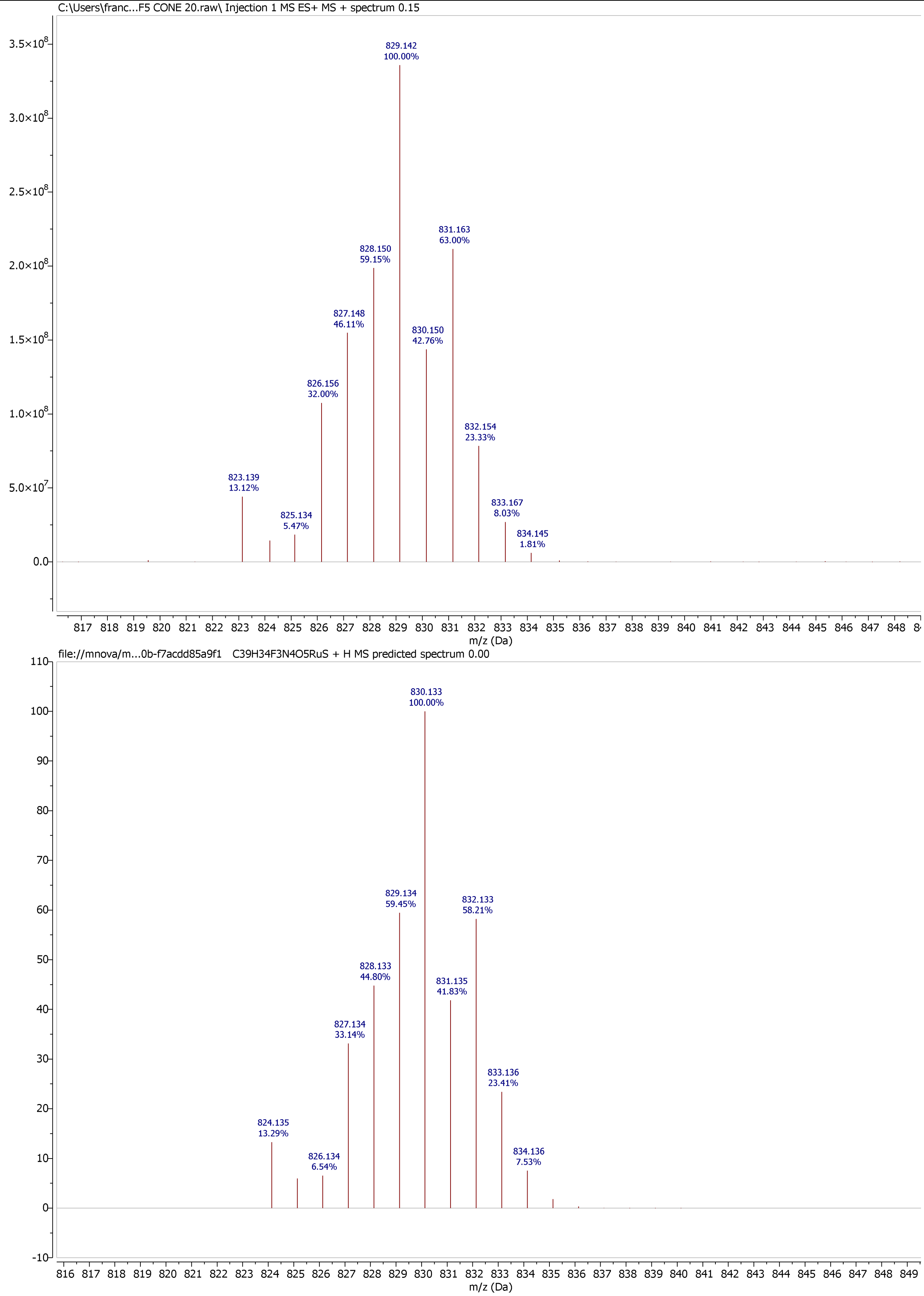

Figure S2. Comparison of the experimental (above) and calculated (below) isotope patterns for key ESI-MS signals for compound C1.

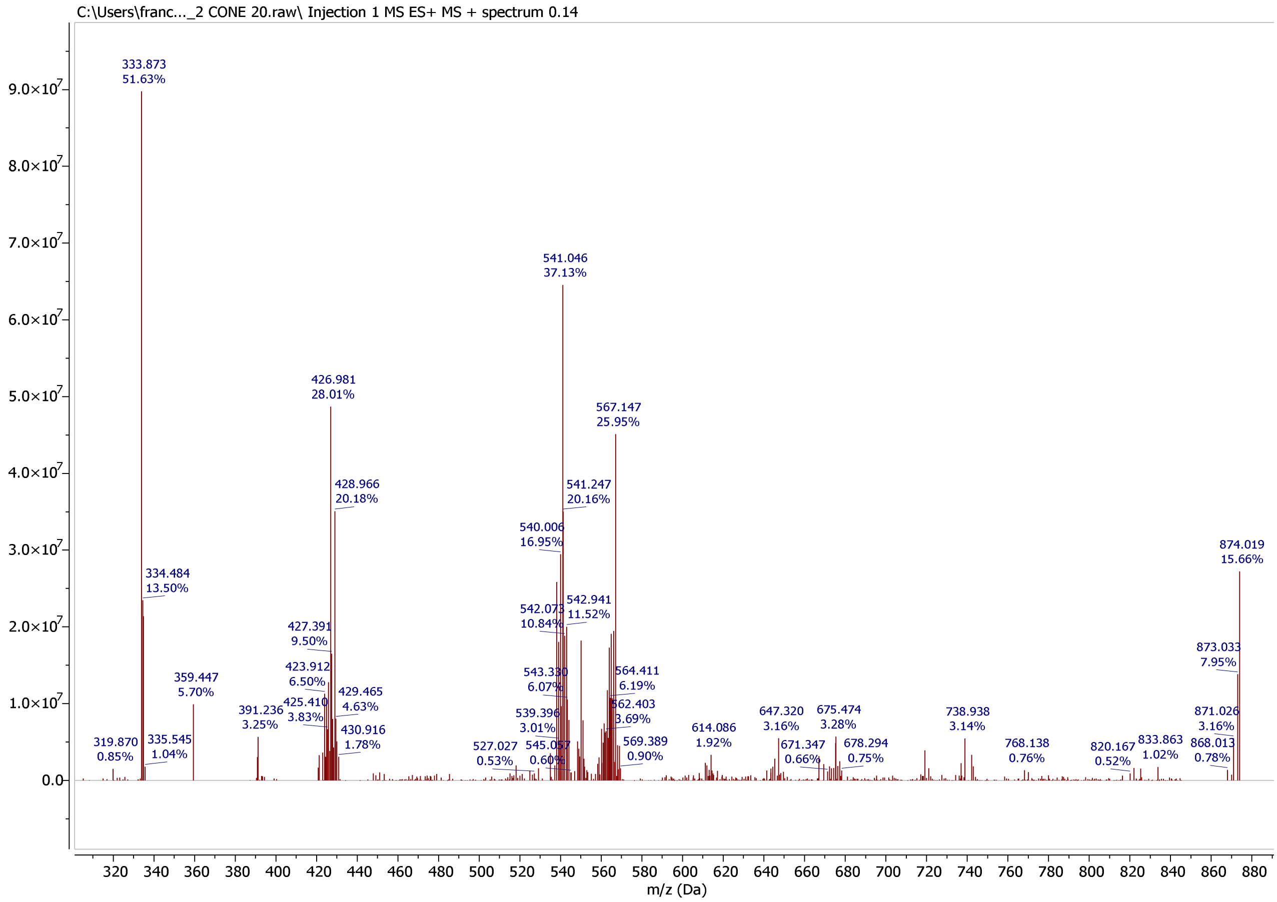

Figure S3. Full ESI mass spectrum of compound C2.

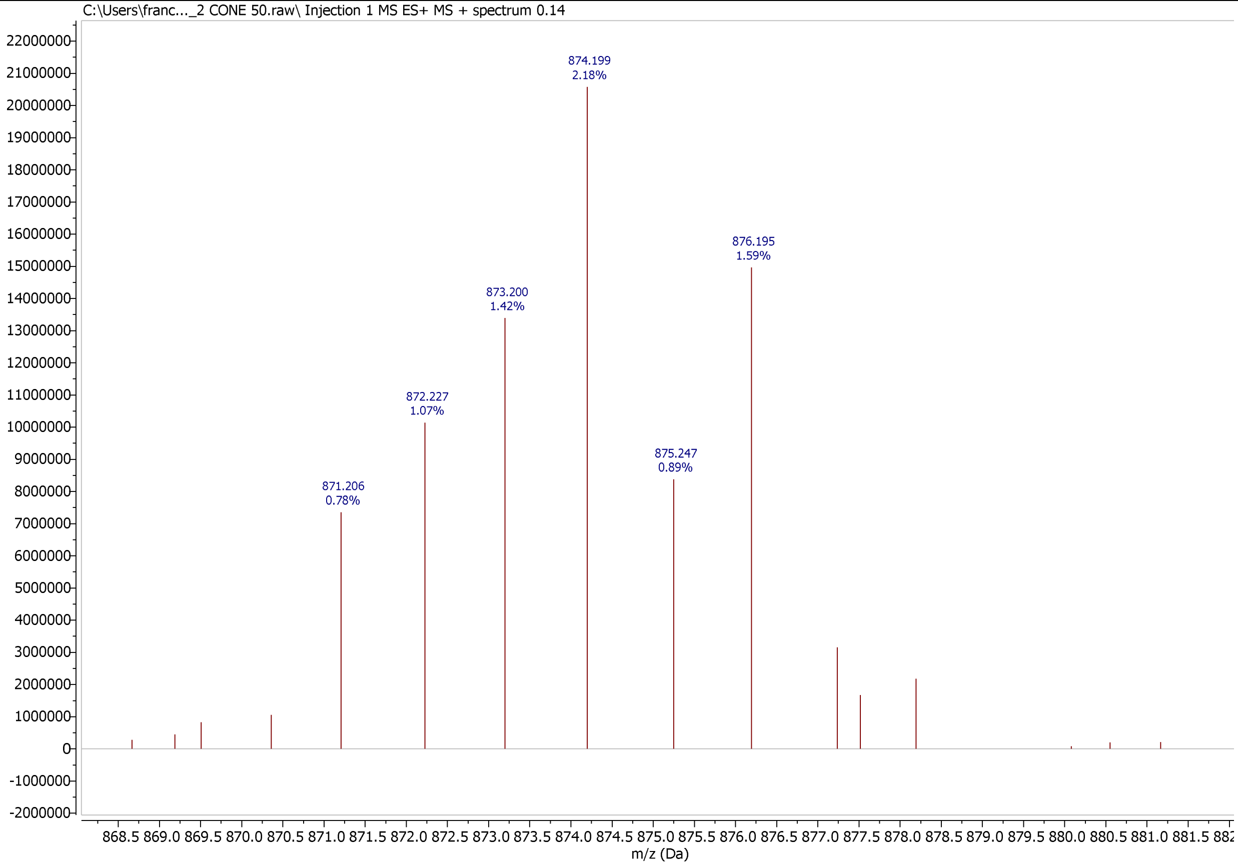

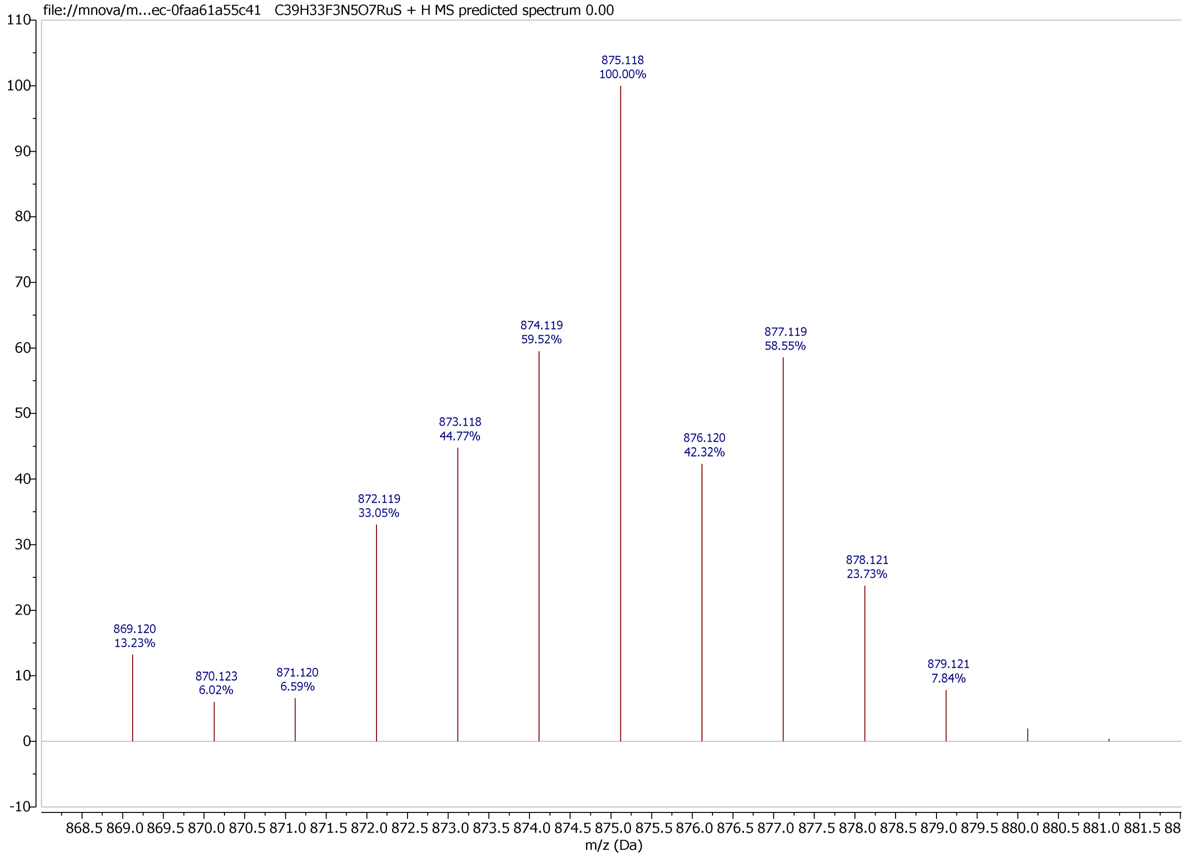

Figure S4. Comparison of the experimental (above) and calculated (below) isotope patterns for key ESI-MS signals for compound C2.

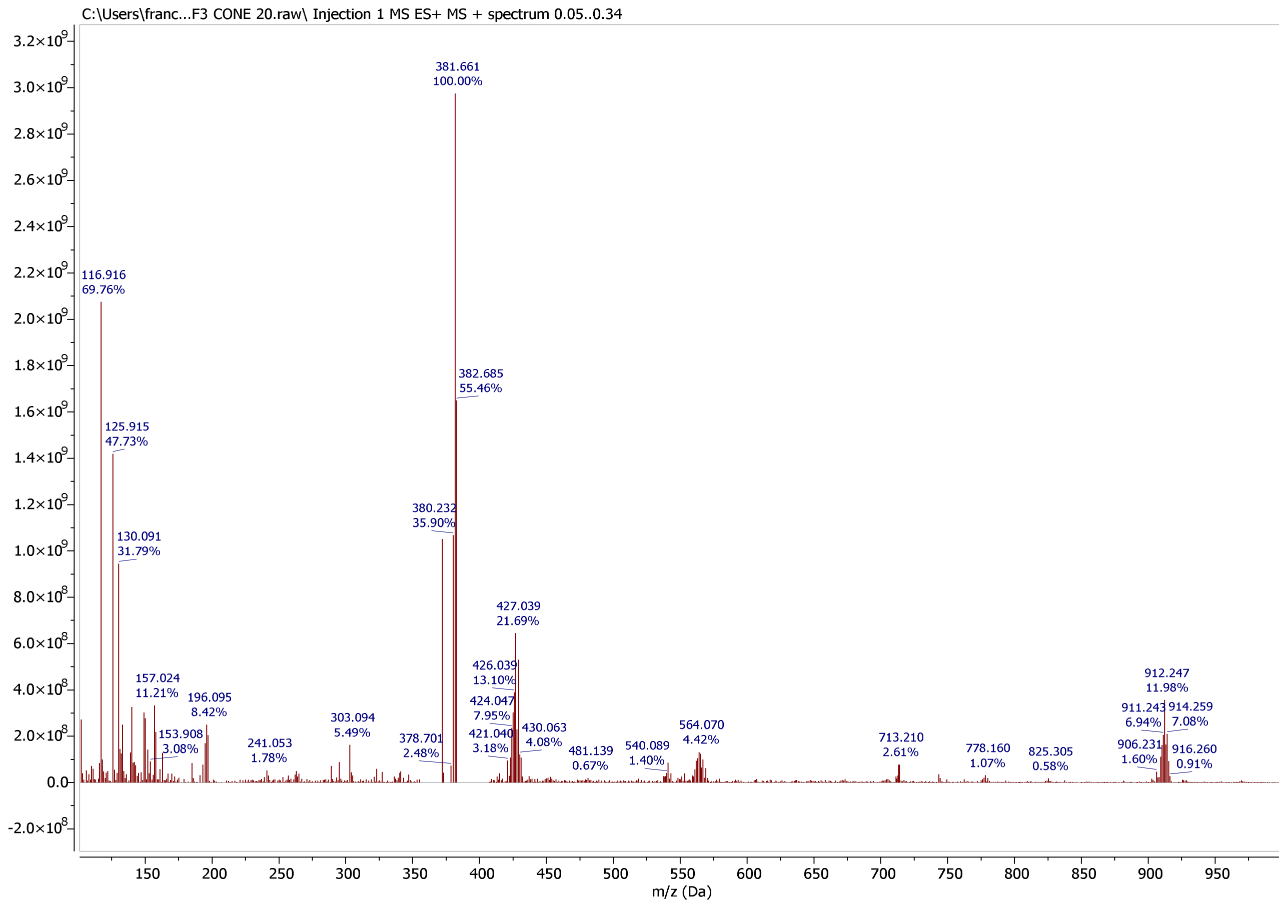

Figure S5. Full ESI mass spectrum of compound C3.

.

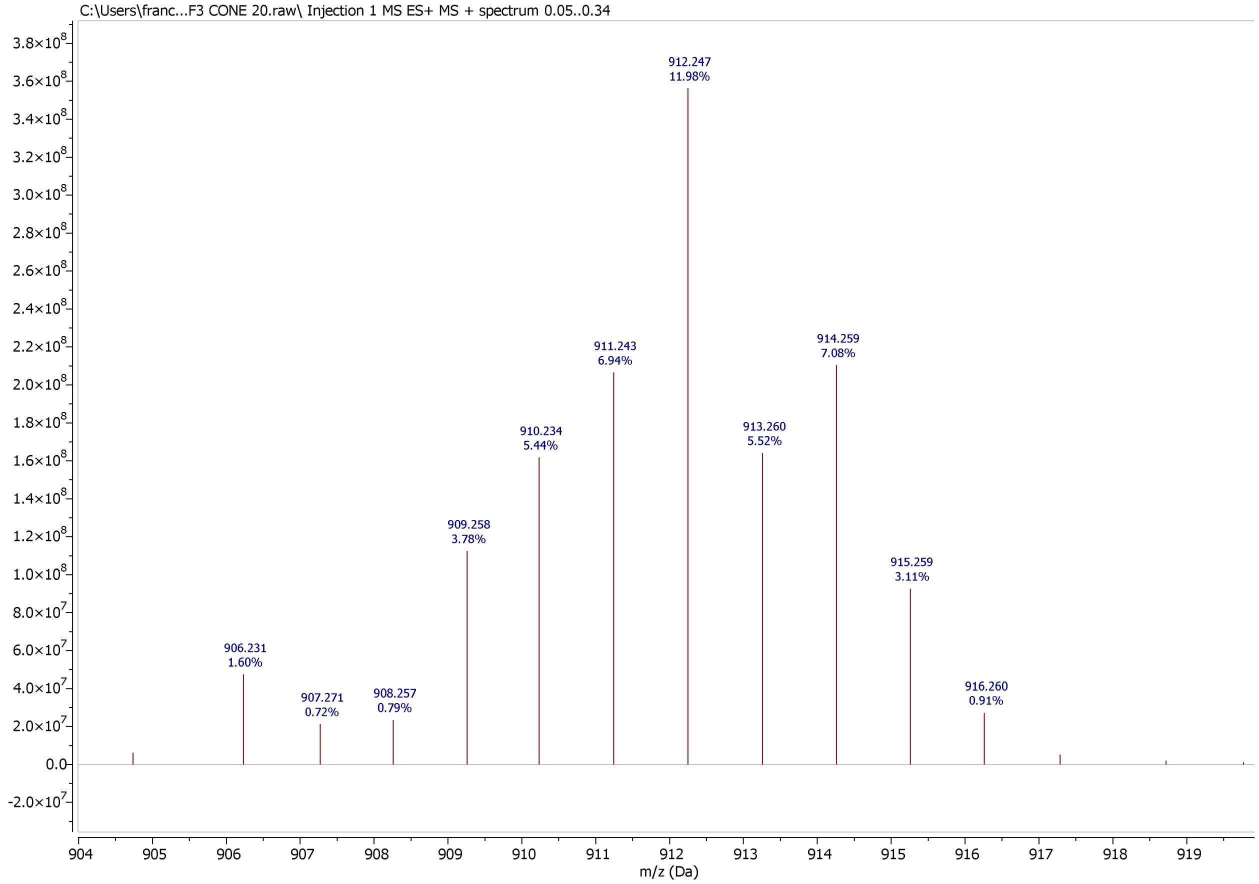
C:\Users\franc...F3 CONE 20.raw\ Injection 1 MS ES+ MS + spectrum 0.05..0.34
912.247
11.98%
911.243
6.94%
914.259
7.08%
910.234
5.44%
913.260
5.52%
909.258
3.78%
915.259
3.11%
906.231
1.60%
907.271
0.72%
908.257
0.79%
916.260
0.91%
m/z (Da)

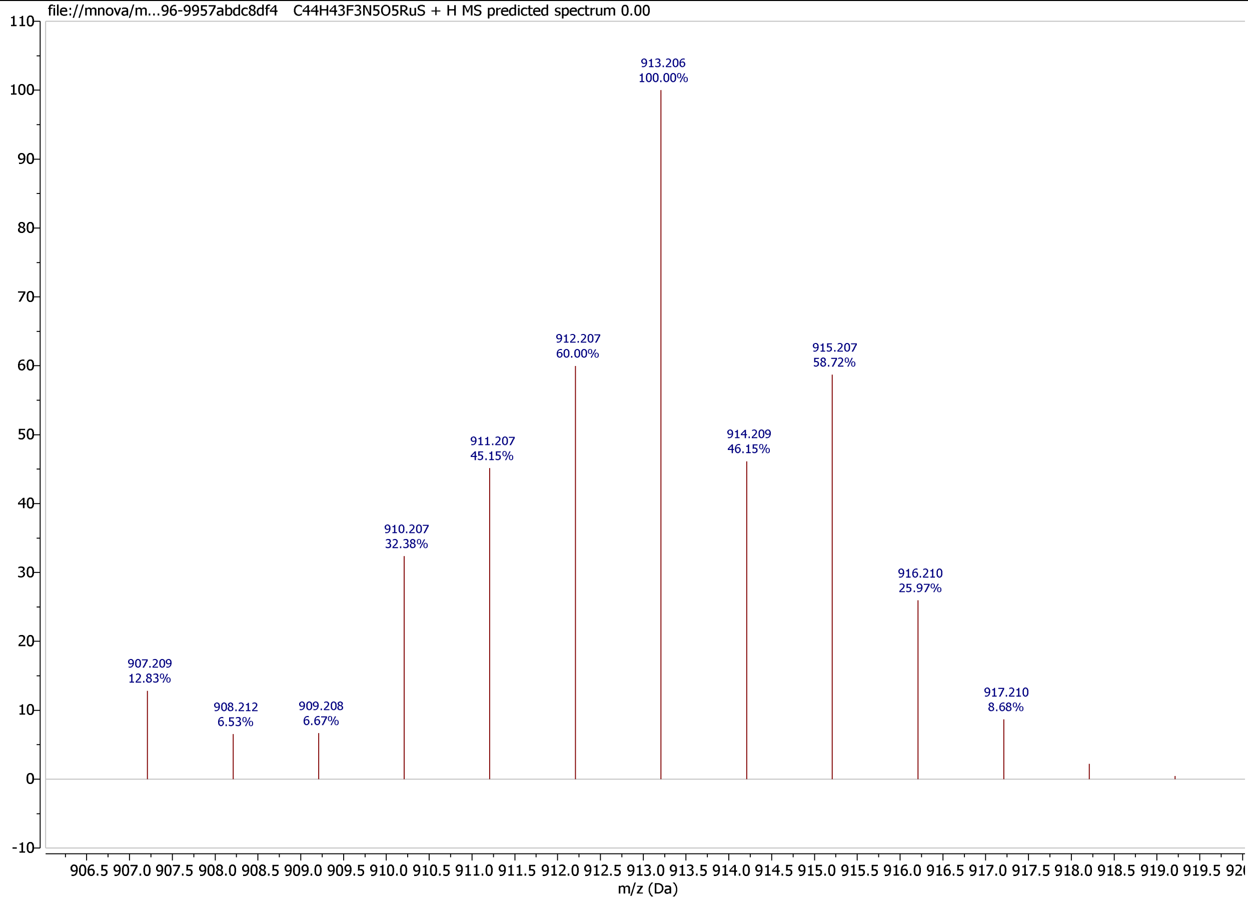

Figure S6. Comparison of the experimental (above) and calculated (below) isotope patterns for key ESI-MS signals for compound C3.

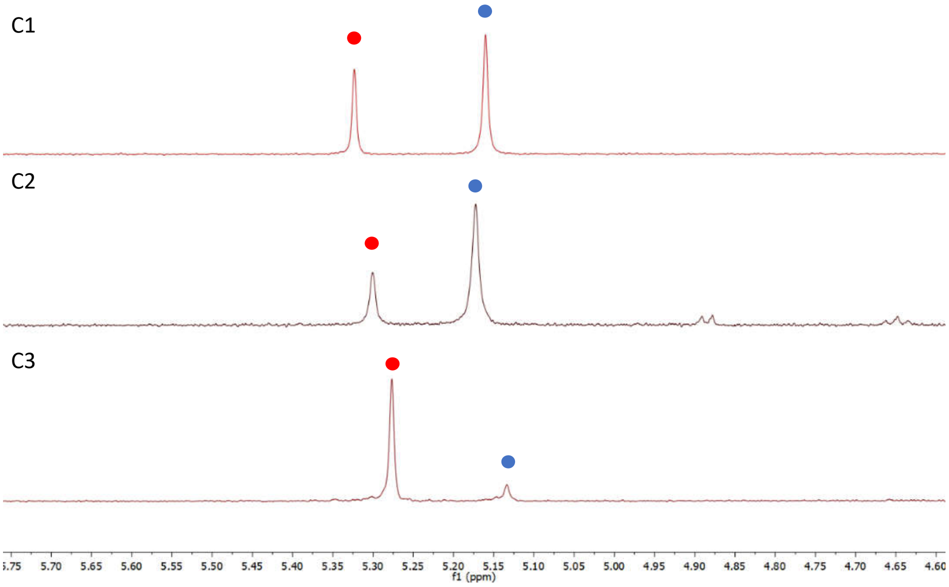

Figure S7. Comparison of the naphthalimide release in C1, C2 and C3 after 3 hours of irradiation.
